# Experiences of working as a cultural doula in Sweden: An interview study

**DOI:** 10.18332/ejm/137365

**Published:** 2021-06-25

**Authors:** Ingrid Ström, Amanda Söderman, Margareta Johansson

**Affiliations:** 1 Department of Women's and Children's Health, Faculty of Medicine, Uppsala University Hospital, Uppsala University, Uppsala, Sweden; 2 Department of Obstetrics and Gynecology, Uppsala University Hospital, Uppsala University, Uppsala, Sweden

**Keywords:** childbirth, cultural doula, foreign-born, interview study, maternity care, support

## Abstract

**INTRODUCTION:**

Cultural doulas have a unique role in providing support to foreign-born women and improving their reproductive health. The aim was to explore cultural doulas’ experiences of giving support to foreign-born women during pregnancy and after childbirth.

**METHODS:**

A qualitative descriptive study with an inductive approach included six cultural doulas in Sweden. Individual semi-structured interviews were conducted, and the data were analyzed with qualitative content analysis.

**RESULTS:**

The theme ‘To lead the way with pride’ was explored and described by the categories: A rewarding mission, A personal guide, and Makes the new society understandable. The cultural doulas gave support in the women’s mother tongue, something they experienced had contributed to reducing the risk of misunderstanding between women and healthcare professionals. The cultural doulas found their work important because the support they provided to women made healthcare more available to them.

**CONCLUSIONS:**

The cultural doulas felt proud when they experienced their work as meaningful and important. It was clear to them that their support and guidance had a positive impact on the women’s reproductive health as well as their integration into Swedish society. Cultural doulas could play an important role in building equal maternal healthcare in Sweden.

## INTRODUCTION

Women born in low-income countries who immigrated to Sweden suffer from a higher risk of maternal death^1^, and their children a higher risk to die during the perinatal period^2^ than women born in Sweden. This higher prevalence seems to be caused by the inability of the healthcare system to meet the needs of these women due to linguistic and cultural barriers. Poor quality in healthcare, such as mistakes in diagnostics, is more common within this group of women^1^. Women born in a low-income country often lack knowledge about the Swedish antenatal care system. They often register late in the pregnancy and show up to fewer planned appointments with the midwife^3^. When foreign-born women do take part in antenatal care, they experience this less inclusive than women in the general population, caused by discrimination from healthcare staff, including lack of information in their mother tongue^4^. The levels of experiencing childbirth-related fears are higher within this group of women^5^. They are more often reported to be confronted with mental health issues than Swedish women in general^6^. Support during pregnancy and childbirth has been proven with positive effects on pregnant women’s mental health and wellbeing^7^.

A doula is a person without medical training involved in a woman’s pregnancy and childbirth and gives emotional, social and psychological support^8^. In Sweden, projects with cultural doulas have been initiated in the last decade. This means that women with varying backgrounds regarding culture and language are employed to support pregnant foreign-born women. The main purpose of the cultural doula is to increase healthcare availability and maternity care to foreign-born women^9^. Foreign-born women that received support from a cultural doula have reported that the cultural doulas provided them with important information, increased their trust in Swedish maternity care as well as reduced their feelings of loneliness. Even midwives have expressed a positive experience since the cultural doula facilitates communication between the foreign-born women and themselves^10^. Cultural doulas have a unique role in providing support to foreign-born women and contributing to their reproductive health. There has been little documentation about how the cultural doulas experience their work, hence this study sought to explore cultural doulas’ experience of giving support to foreign-born women during pregnancy and after childbirth.

## METHODS

### Study design

A descriptive qualitative methodology with an inductive approach^11^ was adopted and the research process followed the RATS guidelines^12^.

### Setting and participants

The participants were recruited by advertisement in 2018 from the Swedish project Cultural Doula in the County of Uppsala. The project was carried out between the years of 2018 and 2020 with the ambition to improve the maternity healthcare received by foreign-born pregnant women with limited social support. Foreign-born women in this article refers to women who were born in a country outside of Scandinavia and arrived in Sweden less than five years ago. The cultural doula provided support to the woman during the pregnancy and postpartum period but could not support the women during childbirth due to economic limitations within the project. When an antenatal care midwife identified pregnant women that could benefit from a cultural doula, information about the project Cultural Doula was given. If the woman approved, the cultural doulas were matched to each woman’s language knowledge. The midwife passed on contact details to the project, which matched the woman with a cultural doula that could speak the same language. The doulas were intended to meet each woman 3–4 times, with one follow-up meeting postpartum. The length of time each meeting took was individual, and mostly they kept contact with each other by phone in between, which was not implied by the project manager but voluntary. The encounters between the cultural doula and the women often took place in public places or municipality areas. The municipality organizes social gatherings, free of charge, for young children and parents, a place the cultural doulas introduced to the women. During 2018 and 2019, the cultural doulas in total supported 197 women.

The criteria for selecting representative cultural doulas in this study was that they were fluent in either Swedish or English and at least one of the languages: Amharic, Arabic, French, Kurdish, and Tigri. Another inclusion criterion was that they had participated in an education provided within the project. The education consisted of eight days and included subjects related to pregnancy, childbirth, and the Swedish Healthcare System. To participate in the study, the cultural doulas were also required to have previous experience in giving support to pregnant women in the role as a cultural doula, however, no lower limit was set. Information about the study and an invitation to participate was sent out by email through the project manager, to all cultural doulas within the project (n=12). Seven of the cultural doulas agreed to participate in the study, but one did not respond to the official invitation. In total, six cultural doulas were interviewed.

### Measures and variables

**Figure 1:**
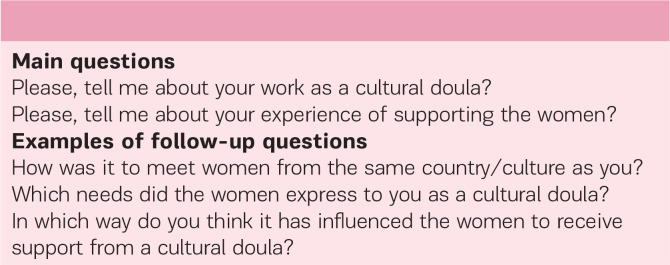
Questions in the interview guide

Individual audio-recorded semi-structured interviews were conducted. One face-to-face and five over the telephone, as preferred by the participants. The participants were interviewed in Swedish, except one in English, and they lasted between 30 and 45 minutes, with an average of 35 minutes. The questions asked were followed by an interview guide, and the interviews were carried out in a friendly atmosphere to get detailed descriptions of the phenomena under study (Figure 1).

### Data analysis

Qualitative content analysis with an inductive approach following Graneheim and Lundman’s description^11^ was used. The interviews were transcribed verbatim, and the two first authors checked each other’s transcripts for quality assurance. The transcripts were then read and re-read to give a deeper understanding of their content, and looked at repeatedly as the work proceeded. The data were condensed into shorter titles, and statements that related to the same overall category were identified and labelled. The statements put forth by the doulas were sorted into three categories that further explored the overall theme: ‘To lead the way with pride’. During the data analysis, the evolving categories and themes were discussed frequently between the three authors. Last author has extensive experience of qualitative research, something that facilitated and improved the quality of the analysis. All authors had clinically experience of healthcare as registered nurses; the two first authors had experiences as midwifery students and the last author extensive experience of clinical midwifery work and midwifery education.

### Ethical considerations

All the participants received written and oral information about the study, that participation was on a voluntary and confidential basis. Verbal consent was obtained, and no identifiable information about the participants is reported. This study was conducted initially as a Master’s thesis within the Midwifery programme at Uppsala University. Therefore, According to Swedish Law, it does not require ethical approval by any Human Research Ethics Committee. However, the study plan was approved by Uppsala University and the manager for the cultural doula project.

## RESULTS

### Study participants

Six women within the project Cultural Doula in the County of Uppsala participated, aged 30–65 years. They originated from countries in the Middle East and northeast Africa, and had lived in Sweden between 5 and 43 years.

### To lead the way with pride

The data analysis explored the theme ‘To lead the way with pride’, reflecting the cultural doulas experiences about their work. The cultural doulas qualifications within language, culture, society, and the Swedish healthcare system provided them with the needed skills to lead the way for the women they guided. When realizing that their work positively impacted women’s lives, they felt proud to work as a cultural doula. The overall theme was then deepened in its meaning by the categories: A rewarding mission, A personal guide, and Makes the new society understandable.

### A rewarding mission

The cultural doulas experienced their work as being meaningful because they could clearly see that their support made a difference for the women. With the support of the cultural doulas, the women were able to become more involved in society and increased their mental wellbeing. They also made new friends and dared to open up more to their midwives, something that made them feel proud to be able to achieve. Seeing the immense impact of the support the cultural doulas were able to provide, made them all want to continue working as cultural doulas:

*‘It's a very good experience. I'm very happy with my work as I have a chance to make a difference and to make other peoples' lives easier.’* (Cultural doula No. 4)

### A personal guide

The cultural doulas’ knowledge within language, culture, society, and the healthcare system, both elsewhere and in Sweden, were elements that made their work successful. Because of the women’s different backgrounds, the support needed to be adapted and varied from woman to woman, and the cultural doula had to be flexible and adjust the support to each individual.

Relationships took time to build, and the women developed confidence for the cultural doula over time. The cultural doulas had personal experience of being newly arrived in Sweden, something that helped them in their work with women. They recognized the situation the women were in and knew what was important to know and how to assist:

*‘I feel for them … because, when I came to Sweden, I got pregnant right away! I actually needed someone that could help me because everything was new to me language, society, the healthcare system, so there is a big difference [between my home country and here]. I actually understand what they need help for.’* (Cultural doula No. 3)

The cultural doulas gave information and prepared the women for childbirth, something many women felt worried about. The cultural doulas experienced their support could reduce the anxiety regarding childbirth among the women. The cultural doulas could also identify misunderstandings; one example was normalization concerning decreased foetal movement:

*‘Also, it reduces the risk for them. Sometimes there are women that think it is normal that the baby is not moving in the stomach. I inform them that it's not normal, you should contact your midwife or the maternity ward.’* (Cultural doula No. 6)

### Makes the new society understandable

The cultural doulas guided women into the new society and experienced they could increase the women’s trust in the Swedish society and the healthcare system. The women were often suspicious of the Swedish society, and the cultural doulas could deny false rumors and provide true information, which in turn increased the women’s trust in the society as well as healthcare.

*‘They start with being suspicious, “Why me? Why didn't they tell my friend who is pregnant to have a doula?” So we try to explain … Because many women think that we are coming from the Social Services.’* (Cultural doula No. 6)

The cultural doulas experienced that the women they met often felt lonely and had limited social networks in Sweden. The support from the cultural doulas helped reduce the feeling of loneliness, as the women felt supported. The cultural doula also contributed to increase the women’s own social networks by introducing them to social contexts run by the municipality where they could meet new friends in a similar situation as their own:

*‘How happy they [the women] are that they can come [to …] and meet others… a midwife will come, they can ask many private questions too, and they dare to do it! So that makes me happy.’* (Cultural doula No. 1)

## DISCUSSION

In this study, the cultural doulas were proud of their work as it was rewarding to experience that their support could make a difference for the foreign-born women they met. The cultural doulas could often support the women in their mother tongue, something the cultural doulas experienced contributed to reduced risk of misunderstandings between the women and the healthcare professionals. Language barriers are a proven risk for receiving suboptimal care within the group of foreign-born women^1^, strengthening the cultural doulas experience of their work, as they could interpret various situations for the women, both linguistically and culturally. Foreign-born women have also expressed less satisfaction with maternity care than native-born women, which is often caused by lack of information in their mother tongue. These negative experiences have resulted in feelings of loneliness, of being unsafe, and fear of receiving deficient care^4^. Akhavan et al.^10^ has reached the conclusion that foreign-born women supported by a cultural doula during pregnancy experienced being given important information that would otherwise have been inaccessible to them, which strengthened their trust in Swedish healthcare system. Foreign-born women encounter a higher risk of maternal^1^ and perinatal death^2^. During the interviews, it emerged that women were unaware that decreased foetal movement is an observandum, something that the cultural doulas could inform about. This could in the long-term, have an impact on the perinatal deaths within this group. The cultural doula could also function as a link between the women and the healthcare staff, as they guided the woman to the right instance, depending on the topic brought up by the woman. The greater understanding and accessibility of the existing healthcare system, the more significant number of visits to the antenatal clinic, thus creating a chance of improved health during pregnancy. The cultural doulas in this study registered that they contributed to a better mental health for women by reducing loneliness, through support as well as introducing social places within the municipality. This made it easier for the women to integrate in the society, which increased their social network. To feel integrated in a society is important, since increased ability to take part and make an impact in one’s life has great health benefits, for both the women themselves and their children^13^. Foreign-born women in Sweden are reported with poor mental health^6^, which highlights the importance of supporting this group. The cultural doulas also experienced they could reduce anxiety and fear related to childbirth, something that has been shown more common in this group^5,10^. Another Swedish study implies that cultural doulas that attend the women’s childbirth are highly appreciated by the women, as they can interpret and create a safe environment^14^. The cultural doulas in this study experienced that woman easily turned to them for support, mainly due to the doulas speaking the same language as the women, combined with their valuable knowledge of the Swedish culture and the healthcare system. The cultural doulas shared their information with them, illustrating their unique role in supporting the pregnant foreign-born women.

### Strengths and limitations

To our knowledge, this is one of the first studies to examine cultural doulas’ experiences of supporting foreign-born women during pregnancy and postpartum. However, a limitation of our study is the small number of participants and the findings should be transferred with caution. The description of the phenomena started to repeat in the last interviews, which indicated saturation and trustworthiness. Strength of our study is that all interviews were carried out by the two first authors together, following an interview guide, and a joint data analysis including all authors.

## CONCLUSIONS

The cultural doulas felt proud when they experienced their work as meaningful and important. It was clear to them that their support and guidance had a positive impact on the childbearing women’s reproductive health as well as their integration into Swedish society. This study clarifies the importance of cultural doulas becoming a natural part of maternity care. Their work can be a step towards an equal maternal healthcare in Sweden.

## Data Availability

The data supporting this research cannot be made available for privacy reasons.
